# Implementing of Active Brain-Dead Donor Identification Strategy in a Single Donor Center: One Year Experience

**DOI:** 10.3390/medicina56080366

**Published:** 2020-07-22

**Authors:** Akvilina Trilikauskienė, Irena Maraulaitė, Diana Damanskytė, Dovilė Lukminaitė, Neringa Balčiūnienė, Tomas Tamošuitis

**Affiliations:** 1 Kauno Klinikos Neurosurgery Department, The Hospital of Lithuanian University of Health Sciences, 50161 Kaunas, Lithuania; irena.maraulaite@gmail.com (I.M.); ddamanskyte@gmail.com (D.D.); neringa.balciuniene@kaunoklinikos.lt (N.B.); tomas.tamosuitis@kaunoklinikos.lt (T.T.); 2 Kauno Klinikos Emergency Medicine, The Hospital of Lithuanian University of Health Sciences, 50161 Kaunas, Lithuania; dovile.lukminaite@kaunoklinikos.lt

**Keywords:** organ donation, brain dead donors, donor identification

## Abstract

*Background and objectives:* Organ shortage is considered to be a major limitation for increasing transplantation rates. Brain-dead donors (DBDs) are an important source of organs, but up to 50% of potential DBDs might not be identified. An active brain-dead donor search could potentially increase a deceased donor pool. The aim of this study was to evaluate the effectiveness of an active potential DBD identification program and to evaluate one year impact on the potential organ donor pool in Lithuania‘s biggest medical institution. *Materials and Methods:* An organ donor coordinator service was established and active DBD search strategy was implemented in the hospital of LSMU Kauno Klinikos, and retrospective data analysis was performed between December 2016 and December 2017. Collected data was compared to the available data of the previous year in the same center and to the donation dynamics of the whole country. *Results:* A total of 6734 patients were treated in all intensive care units (ICU), and 234 (3.5%) of them were identified as possible donors. No increase in potential donor’s number was observed in study year (*n* = 34) compared to remote year (*n* = 37). No significant difference in potential donor’s demographic data, cause of death, family refusals and medical contraindication rates. Cerebral angiography (CA) repeated in 20% of potential donors in order to confirm brain death diagnosis. More potential donors for whom CA was repeated had decompressive craniectomy done (66.7% vs. 33.3%, *p* = 0.018). Decompressive craniectomy statistically significantly increases the rate of repeated CA (OR 12.7; 95% CI, 1.42–113.37; *p* = 0.023). Active search strategy increased length of hospital stay of potential donors comparing to previous year (3.97 ± 4.73 vs. 2.51 ± 2.63, *p* = 0.003). An optimal time of the first four days of hospitalization to identify a potential donor was observed during our study (OR 10.42; 95% CI, 4.29–25.34; *p* = 0.001). *Conclusions:* We were not able to demonstrate active donor identification strategy superiority over the passive strategy during a short one year period; nevertheless, valuable knowledge was gained in brain death diagnostics, new terminology was implemented, and the stability of actual donor numbers was observed in the experimental donor center in the light of decreasing national results. Long-term strategy is required to achieve sustainable results in organ donation.

## 1. Introduction

Organ transplantation became the optimal treatment method for terminal organ failure in the last 50 years, as it improves quality of life and is cost-effective [1,2,3,4]. Organ deficiency is considered a major limitation in increasing transplantation rates and results in lengthening transplant waiting list and 11–20 people’s death on that list every day because of organ shortage [4,5,6].

Deceased donors, either brain-dead donors (DBDs) or donors after circulatory death (DCD), are considered to be the main organ source for transplantation, but the tendency of reduction of potential DBD donors is being reported [4,7]. This tendency is likely to progress due to improvement in neurocritical care and road safety programs leading to a decrease in patients with devastating brain injury [8]. Alternative strategies such as expanded criteria donors, living donation, and donation after circulatory death or split techniques were developed with limited success to cover this opening gap [9,10,11,12,13,14].

There have been both living and deceased donation programs with ranging results 10–21 donors per 1 million population in the Lithuania within last 15 years. The vast majority of organs are procured after declaration of brain death. Living donation and recently introduced DCD programs have a limited impact; however, their potential is great [15,16]. According to Lithuanian National Transplant Bureau, 408 patients were on a transplantation waiting list, and only 142 organs from 101 potential donors were transplanted in 2017. For several years, the tendency of dropping potential DBD numbers has also been observed in the biggest donor and transplant center of Lithuania [16]. It is important to improve the process of deceased donation, and a pro-active donor identification strategy seems to be beneficiary [17,18,19].

Although identification is the first and most important step of donation process, up to 50% of potential donors still might not be identified [20,21,22,23,24,25]. The identification process consists of healthcare professionals’ ability to assess clinical triggers of potential donors and to refer them to a donor coordinator. Squires et al.’s [21] systematic review showed that neurologic, medical decision, cardiorespiratory, and administrative criteria can be used for deceased organ donation identification and referral. Low score of the Glasgow Coma Scale (GCS) in a patient with a devastating brain injury is the most commonly used trigger to refer potential donors (PDs) [4,26]. Various countries implement new methods to improve the identification process. Beigee et al. [17] assessed that more proactive strategies for brain-dead donor detection significantly increase the donor pool. They upgraded their identification strategy and improved results by increasing phone calls and the inspections number to the ICU for one year. Ludwig et al. [22] concluded that a computerized scale for the active search for potential donors was also effective. A computerized scale based on Sepsis-Related Organ Failure Assessment (SOFA) and Acute Physiology and Chronic Health Evaluation II (APACHE II) prognostic indexes was applied as an instrument for organizing the organ donation and transplantation process in the hospital of the study. Zier et al. [27] advocated for an electronic clinical decision support system to improve organ donation. They implemented an electronic system that automatically notified their organ procurement organization of patients meeting clinical triggers indicating brain death.

The aim of this study was to evaluate the effectiveness of an active potential DBD identification program and to evaluate the one-year impact on the potential organ donor pool in Lithuania‘s largest medical institution.

## 2. Materials and Methods

The donor coordination team was implemented in the Hospital of Lithuanian University of Health Sciences (LSMU) Kauno klinikos between December 2016 and December 2017. Retrospective analysis of donor coordination team data was performed. The study was approved by the Kaunas Regional Biomedical Research Ethics Committee (approval code: BE-2-43; approval date: 2 June 2020).

The donor coordination team, composed of five ICU physicians with neuro ICU backgrounds, was set up prior our study period. No system of active possible DBD search was available before study period in the hospital. A donor coordinator visited five specialized ICUs on a daily basis—neurosurgical, cardiosurgical, general, trauma, and cardiologic ICUs with total bed number of 81. Patients with severe neurological damage were referred by the treating physician to the donor coordinator. Neurological assessment was carried out for every selected patient by coordinator and patients who met possible brain-dead donor criteria—1) severe brain injury and 2) GCS ≤ 5—were identified. All data was registered in an original color-coded follow-up system according to the patient status. Three main colors were used—1) green was used for active follow-up with recorded trend of GCS; 2) yellow was used when the patients were put on hold because of sedation; 3) red color marked the completion of the follow-up. The result of the completion was circulatory death, recovery, or brain death (Table 1). Collected and possibly comparable data were compared to the matching period of the previous year results in the study center and in the whole country. Only potential and actual donor data were compared with the data from the previous year, since no possible donor data were collected previously.

Definitions of the organ donor groups are based on national protocols published by the Lithuanian Health Ministry. A potential DBD donor is a patient who fulfils clinical brain death criteria and whose protocol for brain death is started. An actual DBD donor is a potential donor from whom at least one organ was recovered for the purpose of transplantation. The definition of possible donor is not mentioned in the Lithuanian legislation. According to the WHO, a possible DBD donor is considered a patient in coma with devastating brain injury or lesion and apparently medically suitable for organ donation. A possible DBD definition was included in our study. In Lithuania, brain-death protocol is initiated when all six brainstem reflexes (pupillary, corneal, oculovestibular and oculocephalic, cough and gag, pain stimuli) and spontaneous breathing are clinically absent. Brain death is determined clinically by three physicians (two intensivists and a neurologist or neurosurgeon) every 12 h for three times. Brain death diagnosis can be determined at any time after the first brain death confirmation is clinically determined by performing ancillary tests (cerebral angiography, computed tomography angiography (CTA), electroencephalogram (EEG)) if available in hospital. In our study, cerebral angiography was performed in every case to confirm brain death. Clinical and demographic data was recorded for all potential brain-dead donors on the registry.

Statistical analysis was performed with the SPSS Version 23.0 statistic software package (IBM Corp., Armonk, NY, USA). A value of *p* < 0.05 was considered statistically significant. Statistical analysis used the chi-squared test or Fisher’s exact test for categorical variables and the Mann–Whitney test for comparisons of means.

## 3. Results

**Figure 1:**
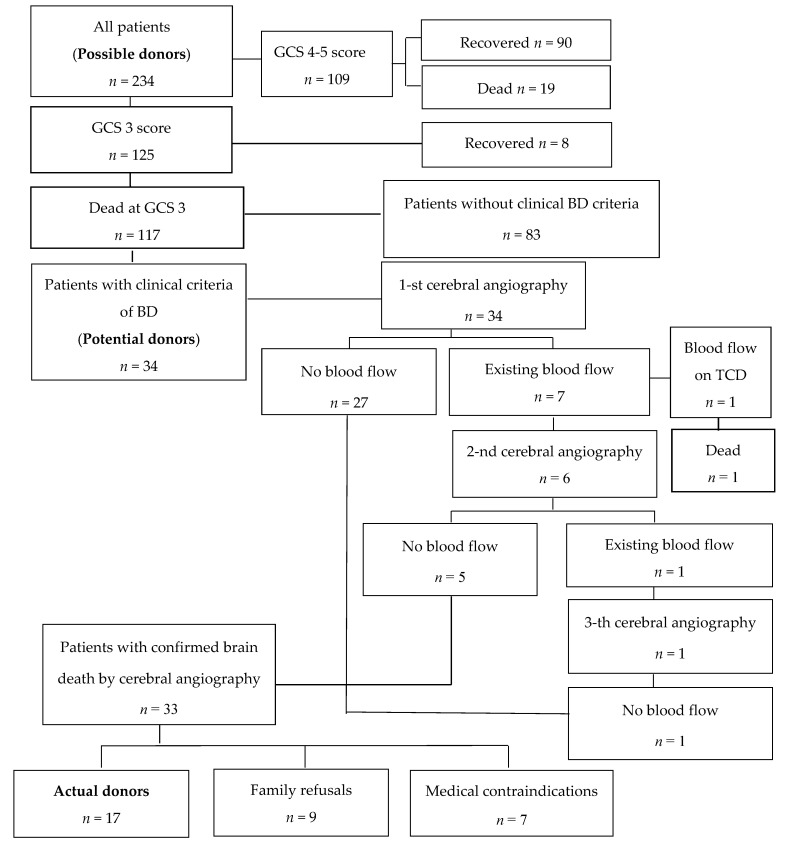
Possible donor data.

During the study period, a total of 6734 patients were treated in all ICUs; 234 (3.5%) of them were identified as possible donors (met our criteria: were GCS ≤ 5 and had severe brain injury). In addition, 117 of 125 GCS 3 patients died during the hospitalization period, and three survived ICU stay and were transferred to wards having been tracheotomized and with spontaneous breathing. Most of deceased GCS 3 patients (83 out of 117) died after cardiac arrest not fulfilling BD criteria (with preserved spontaneous breathing or brain stem reflexes), and 34 were identified as potential DBDs. A flowchart of possible donor data is presented in Figure 1.

No increase in the number of potential donors was observed in the study year compared to the remote year (34 vs. 37). There was no significant difference in potential donor demographic data. The mean age was 52 ± 13.34 in the remote year and 53.41 ± 16.17 in the study year (*p* = 0.728). Males with spontaneous intracerebral hemorrhage (ICH) as the main cause of brain death were dominant in remote and study years (43.2% and 29.4%), and the other causes remained subdural hematoma (SDH) (21.6% vs. 23.5%), subarachnoid hemorrhage (SAH) (21.6% vs. 23.5%), and postanoxic encephalopathy (PE) (8.1% vs. 8.8%), *p* = 0.627. Demographic data and cause of death are compared in Table 2.

**Figure 2:**
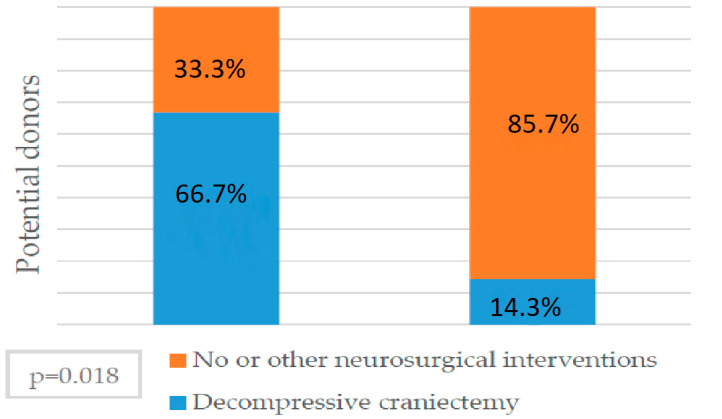
Decompressive craniectomy and repeated cerebral angiography. Impact of decompressive craniotomy on repeated angiography rate. Potential donors with performed decompressive craniotomy have a higher repeated cerebral angiography rate (*p* = 0.018).

Brain death was confirmed for 33 out of 34 potential donors in the study year. Cerebral angiography was repeated twice in six (17.64%) out of 34 cases due to a presented circulation during the first scan and once angiography was repeated for the third time (2.94%). For one of them, cerebral angiography was not repeated because blood flow was found performing control transcranial doppler, and cardiac arrest was registered later before confirming brain death by angiography. The mean time of first cerebral angiography for all potential donors was 138.32 ± 62.51 min after clinical examination confirming brain death. Time of angiography between those with confirmed brain death diagnosis the first time and those requiring second investigation was similar (137.07 ± 64.46 min vs. 144.17 ± 57.48 min). The increased number of potential donors for whom cerebral angiography was repeated had decompressive craniectomy done and was statistically significant (66.7% vs. 33.3%, *p* = 0.018) (Figure 2). Decompressive craniectomy increases the rate of repeated cerebral angiography and is statistically significant (OR 12.7; 95% CI, 1.42–113.37; *p* = 0.023).

Almost one third (29.6%) of all GCS 3 patients had neurosurgical intervention. The surgical intervention rate was higher in the PD group compared to GCS 3 patients with no brain death signs, although no statistical significance was detected (38.2% vs. 26.4%, *p* = 0.355). 

Potential donor conversion to actual donor was similar in both the study and remote years (50% vs. 54.1%, *p* = 0.388). The difference in family refusals between the study and previous years was not significant (26.5% vs. 18.9%, *p* = 0.338). There was also no statistically significant difference between medical contraindications (20.6% vs. 13.5%, *p* = 0.388) and other causes (2.9% vs. 13.5%, *p* = 0.388). Nevertheless, a trend toward negative impact on organ donation was obvious in the study year—the potential donor number decreased by 13% in Lithuania and 8.1% in our center. However, the actual donor number decreased by 27% in Lithuania and by 15% in our center. Comparison of the donation results is shown in Table 3.

In the study year, 2.42 organs were recovered per donor. This number is slightly lower than 2.7 in the previous year.

The length of hospital stay of potential donors was significantly longer in the study period compared to the previous year (3.97 ± 4.73 vs. 2.51 ± 2.63, *p* = 0.003). There is an increased possibility of reporting a potential donor during the first four days of a hospital stay in the study year (OR 10.42; 95% CI, 4.29–25.34; *p* = 0.001).

## 4. Discussion

This is the first attempt to organize a donor-oriented team, an active donor search strategy, and to perform a complex comparative analysis of a DBD pool in a major donor center in Lithuania.

The literature shows that most common trigger for referring possible donors is GCS 8; however, there were new data showing that this trigger is not an adequate predictor [28]. It was decided to start the follow-up of all GCS 5 patients in the ICU because of a high volume of unconscious patients in our large donor center. Fifty percent of the included patients did not deteriorate to GCS 3, and the majority of GCS 5 patients improved in our study. Only 14.5% of possible donors became potential donors. We will change our strategy and start follow-up patients with GCS 3 based on these results as it is suggested in many automated active search systems [28]. Earlier tracking of low-GCS-score patients is probably more related to organ donation as a part of end of life care and active treatment withdrawal, which is not the case in Lithuania. However, many authors suggest informing a coordinator earlier in order not to miss potential donors [28].

Historically, all transplant coordinators were nephrologists in our donor center, but evidence is accumulating that ICU specialists acting as transplant coordinators could improve donation results. For instance, in Spain and Croatia, most of coordinators are ICU specialists, and this is associated with an increased donation rate [28,29]. Intensivist‘s experience in diagnosing brain death and managing of a multiorgan donor is one of the most important elements in the donation process. Therefore, the donor coordination team created in our center consisted of ICU specialists with experience in organ donation and neurocritical care.

The implemented active donor search strategy did not increase the brain-dead donor pool or change the donor profile, and this could be due to the fact that all negative factors for organ donation increased throughout the country during the study period. First of all, a decrease of potential donors was observed in all transplant centers. This is a characteristic swing in organ donation numbers observed but is not explained for the relevant years in Lithuania [30]. This advocates the need for longer observational periods to estimate the impact of various strategies on the potential deceased donor pool. Apart from a decreased number of potential donors, family refusal and medical contraindication for the donation rate went up, causing a drop in actual donor numbers in the country. Family refusal rates have been steadily around 20%–30% over the last decade in Lithuania, despite recent intense public and media campaigns [31]. Nevertheless, increase in family refusal rates was lower in the study center where communication was concentrated within the coordination team. Surprisingly, we were not able to increase the average age of potential/actual donors using an active search, and DBD donors in Lithuania remain of young age compared to the other countries, but with relatively high percent of medical contraindications [19]. This could be explained by the local legislation peculiarity when patients with up-front known medical contraindications for organ donation, e.g., cancer patients being proclaimed brain dead, still have to be registered as potential donors, and this could misrepresent a real percentage of the aborted donation process due to medical contraindications. Nevertheless, we did not find statistically significant difference in all these negative factors comparing the study center donation results with the country’s donation results, yet a trend toward a smaller negative impact was noted, and we can only speculate if these results are influenced by a proactive strategy. A comparison of dynamics in donation results between the study center and the whole country is presented in Table 3.

We found a very high rate of repeated cerebral angiography (CA) after the clinical signs for brain death have been confirmed. CA repeated for 20% of all potential donors with a first test performed in average time just above two hours after clinical examination. Decompressive craniectomy significantly increased the probability of repeated CA in our study, perhaps causing even longer waiting times for brain death confirmation than for the potential donors with intact skulls. It is reported that decompressive craniectomy may lead to false negative results in cerebral angiograms showing normal-appearing blood flow in at least some intracranial blood vessels due to lowered intracranial pressure [32]. Salih et al. [33] showed that, after decompressive craniectomy, brain-death diagnosis determination often exceeded two days, with a mean time 69.4 h. Ancillary tests to confirm brain death after clinical examination are performed in an average mean time of six hours in France and 4.7 h in Canada [34,35]. A CA time limit of six hours has been applied to the local protocol in order to save costs and cut down the amount of contrast used for PD based on our findings.

We found that time from admission to brain death diagnosis was significantly longer in the study period comparing to the previous year, and this could be related to the increased attitude and awareness of personnel of the donation process. These findings could potentially lead to better identification rates of potential donors. We were not able to find any literature reports on this topic.

We would like to highlight a few important findings based on our study that could be implemented at national or regional level. First, there is an urgent need in expanding organ donation terminology according to WHO recommendations by means of the critical pathway [20]. Second, implementing an active possible donor search system in every donor center and collecting and analyzing unified metrics in statistics and quality indicators could improve organ donation results from a long-term perspective. Finally, we would suggest implementing a six-hour time frame for cerebral angiography after clinically determined brain death diagnosis.

### Limitations

We could not analyze how active search impacts the possible donors pool because, before this study, there were no statistical data for possible donors in our hospital. The definition of possible, eligible, or utilized donors was never used in Lithuania.

## 5. Conclusions

An active search of brain-dead donors neither increased the total number of the potential donors nor increased the conversion rates or changed a donor profile in our donor center in a one-year period. A longer observational period and more sophisticated follow-up system might be required. Local protocols were changed in brain death diagnostics, and new terminology was implemented based on the study results. A national long-term strategy is required to strengthen all the components of organ donation.

## Figures and Tables

**Table 1:** Example of the color-coded follow-up system.

Patients	Record Number	Age	Diagnosis	Date of Inclusion	January -01	January -02	January -03	January -04	January -05	January -06	January -07
Patient 1	00001	82	ICH	January -01	Sed.	Sed.	5	5	5	Rec.	
Patient 2	00002	62	SAH	January -02		4	4	3	Dead		
Patient 3	00003	42	SDH	January -03			3	3	3	BD (+)	
Patient 4	00004	22	PE	January -05					4	3	BD (−)

Sed.—sedation, yellow color used; GCS score – green color used; Rec.—recovered, red color used; BD (+)—actual brain-dead donor, red color used; BD (−)—not actual brain dead donor, red color used; ICH—intracerebral hemorrhage; SAH—subarachnoid hemorrhage; SDH—subdural hematoma; PE—postanoxic encephalopathy.

**Table 2:** Potential donors’ basic characteristics.

Data	Time Period		
Potential donor number (*n*)	Remote year	Study year	*p*-Value
	37	34	
Age, mean (range) ± SD	52.19 (19–79) ± 13.34	53.41 (20–81) ± 16.17	0.728
Gender			
Male	19 (51.4%)	20 (58.8%)	0.635
Female	18 (48.6%)	14 (41.2%)	
Cause of death			
Subdural hematoma (SDH)	8 (21.6%)	8 (23.5%)	0.627
Epidural hematoma (EDH)	0	1 (2.9%)	
Subarachnoid hemorrhage (SAH)	8 (21.6%)	8 (23.5%)	
Traumatic SAH	0	1 (2.9%)	
Intracerebral hemorrhage (ICH)	16 (43.2%)	10 (29.4%)	
Stroke	1 (2.7%)	3 (8.8%)	
Anoxic brain injury	3 (8.1%)	3 (8.8%)	
Other	1 (2.7%)	0	

SDH—subdural hematoma; EDH—epidural hematoma; SAH—subarachnoid hemorrhage; ICH—intracerebral hemorrhage.

**Table 3:** Comparison of the donation results.

Data	Study Center			Lithuania	
	Remote Year	Study Year		Remote Year	Study Year
Potential donors	37 (100%)	34 (100%)		115 (100%)	100 (100%)
Actual donors	20 (54.1%)	17 (50%)		59 (50.4%)	43 (43%)
Family refusals	7 (18.9%)	9 (26.5%)	*p* = 0.355	22 (19.1%)	30 (30%)
Medical contraindications	5 (13.5%)	7 (20.6%)		7 (7%)	13 (13%)
Cardiac arrest	5 (13.5%)	1 (2.9%)		27 (23.5%)	14 (14%)

## References

[1] Fitzgerald R.D. (1997). Management of the brain-dead organ donor. Saudi J. Kidney Dis. Transpl..

[2] Grinyó J.M. (2013). Why is organ transplantation clinically important?. Cold Spring Harb. Perspect. Med..

[3] Manyalich M., Guasch X., Gomez M.P., Páez G., Teixeira L. (2013). ODEQUS Consortium. Organ donation European quality system: ODEQUS project methodology. Transplant. Proc..

[4] Council of Europe (2016). Guide to the Quality and Safety of Organs for Transplantation.

[5] American Transplant Foundation Facts and Myths. https://www.americantransplantfoundation.org/about-transplant/facts-and-myths/.

[6] Ferreira L.G., Anastácio L.R., Lima A.S., Touslon Davisson Correia M.I. (2013). Predictors of mortality in patients on the waiting list for liver transplantation. Nutr. Hosp..

[7] Girlanda R. (2016). Deceased organ donation for transplantation: Challenges and opportunities. World J. Transplant..

[8] Domínguez-Gil B., Coll E., Elizalde J., Herrero J.E., Pont T., Quindós B., Marcelo B., Bodí M.A., Martínez A., Nebra A. (2017). Expanding the Donor Pool Through Intensive Care to Facilitate Organ Donation: Results of a Spanish Multicenter Study. Transplantation.

[9] Ojo A.O. (2005). Expanded criteria donors: Process and outcomes. Seminars in Dialysis..

[10] Filiopoulos V., Boletis J.N. (2016). Renal transplantation with expanded criteria donors: Which is the optimal immunosuppression?. World J. Transplant..

[11] Waterman A.D., Morgievich M., Cohen D.J., Butt Z., Chakkera H.A., Lindower C., Hays R.E., Hiller J.M., Lentine K.L., Matas A.J. (2015). Living donor kidney transplantation: Improving education outside of transplant centers about live donor transplantation—recommendations from a consensus conference. Clin. J. Am. Soc. Nephrol..

[12] Emre S., Umman V. (2011). Split Liver Transplantation: An Overview. Transplant. Proc..

[13] Shemie S.D., MacDonald S. (2014). Canadian Blood Services—Canadian Critical Care Society Expert Consultation Group. Improving the process of deceased organ and tissue donation: A role for donation physicians as specialists. CMAJ.

[14] Escudero D., Otero J. (2015). Intensive care medicine and organ donation: Exploring the last frontiers?. Med. Intensiva (English Ed.).

[15] Lietuvos Nacionalinis Transplantacijos Biuras Prie LR SAM. http://ntb.lrv.lt/lt/statistika/donoryste.

[16] Tamošuitis T., Maraulaitė I., Albavičiūtė D., Narakaitė A., Balčiūnienė N. (2017). Impact of new organ donation model to the potential deceased donor pool in the Kaunas donor center. Health Sci..

[17] Beigee F.S., Mohsenzadeh M., Shahryari S., Mojtabaee M. (2017). Role of more active identification of brain-dead cases in increasing organ donation. Exp. Clin. Transplant..

[18] Salim A., Berry C., Ley E.J., Schulman D., Desai C., Navarro S., Malinoski D. (2011). In-house coordinator programs improve conversion rates for organ donation. J. Trauma - Inj. Infect. Crit. Care..

[19] Matesanz R., Domínguez-Gil B., Coll E., Mahíllo B., Marazuela R. (2017). How Spain Reached 40 Deceased Organ Donors per Million Population. Am. J. Transplant..

[20] Domínguez-Gil B., Delmonico F.L., Shaheen F.A., Matesanz R., O’Connor K., Minina M., Muller E., Young K., Manyalich M., Chapman J. (2011). The critical pathway for deceased donation: Reportable uniformity in the approach to deceased donation. Transplant International..

[21] Squires J.E., Coughlin M., Dorrance K., Linklater S., Chassé M., Grimshaw J.M., Shemie S.D., Dhanani S., Knoll G.A. (2018). Criteria to identify a potential deceased organ donor: A systematic review. Crit. Care Med..

[22] Ludwig É.F., dos S.B., Pereira M.C.A., Martinez Y.D.É., Mendes K.D.S., Rossaneis M.A. (2017). Prototype of a computerized scale for the active search for potential organ donors. Rev. Lat. Am. Enfermagem..

[23] Ehrle R. (2006). Timely Referral of Potential Organ Donors. Crit. Care Nurse..

[24] Etheredge H.R., Penn C., Watermeyer J. (2018). A Qualitative Analysis of South African Health Professionals’ Discussion on Distrust and Unwillingness to Refer Organ Donors. Prog. Transplant..

[25] Siminoff L.A., Gardiner H.M., Wilson-Genderson M., Shafer T.J. (2018). How Inaccurate Metrics Hide the True Potential for Organ Donation in the United States. Prog. Transplant..

[26] Westphal G.A., Garcia V.D., Souza R.L., Franke C.A., Vieira K.D., Birckholz V.R., Machado M.C., Almeida E.R., Machado F.O., Costa Sardinha L.A. (2016). Guidelines for the assessment and acceptance of potential brain-dead organ donors. Revista Brasileira Terapia Intensiva..

[27] Zier J.L., Spaulding A.B., Finch M., Verschaetse T., Tarrago R. (2017). Improved Time to Notification of Impending Brain Death and Increased Organ Donation Using an Electronic Clinical Decision Support System. Am. J. Transplant..

[28] Sánchez-Vallejo A., Gómez-Salgado J., Fernández-Martínez M.N., Fernández-García D. (2018). Examination of the brain-dead organ donor management process at a Spanish hospital. Int. J. Environ. Res. Public Health..

[29] Busic M., Lovrencic-Huzj A., Randhawa G. (2012). Action Taken to Boost Donor Rate in Croatia. Organ Donation and Transplantation—Public Policy and Clinical Perpectives.

[30] Donation National Transplant Bureau under the Ministry of Health. https://ntb.lrv.lt/en/statistics/donation-1..

[31] Donorystė|Nacionalinis Transplantacijos Biuras Prie Sveikatos Apsaugos Ministerijos. https://ntb.lrv.lt/lt/statistika/donoryste..

[32] Kramer A. (2015). Ancillary Testing in Brain Death. Semin. Neurol..

[33] Salih F., Finger T., Vajkoczy P., Wolf S. (2017). Brain death after decompressive craniectomy: Incidence and pathophysiological mechanisms. J. Crit. Care..

[34] Kerhuel L., Srairi M., Georget G., Bonneville F., Mrozek S., Mayeur N., Lonjaret L., Sacrista S., Hermant N., Marhar F. (2016). The optimal time between clinical brain death diagnosis and confirmation using CT angiography: A retrospective study. Minerva Anestesiol..

[35] MacDonald D., Stewart-Perrin B., Shankar J.J.S. (2018). The Role of Neuroimaging in the Determination of Brain Death. J. Neuroimaging..

